# Cardiovascular magnetic resonance worldwide: A global commitment to cardiovascular care

**DOI:** 10.1016/j.jocmr.2025.101842

**Published:** 2025-01-29

**Authors:** Carlos E. Rochitte

**Affiliations:** a Heart Institute, InCor, University of São Paulo Medical School, São Paulo, SP, Brazil; b Hcor, Heart Hospital, Associação Beneficiente Síria, São Paulo, SP, Brazil

Our community has made remarkable strides in establishing cardiovascular magnetic resonance (CMR) as a cornerstone of modern cardiology, but our journey is far from over. As the incoming President of the Society for Cardiovascular Magnetic Resonance (SCMR), I am filled with gratitude, purpose, and responsibility. My vision for the year ahead is centered on addressing key challenges and seizing opportunities to ensure that the benefits of CMR reach patients worldwide.

CMR has established itself as a critical tool in the diagnosis and management of a wide range of cardiovascular conditions. Its ability to provide non-invasive, high-resolution imaging without ionizing radiation makes it uniquely valuable in assessing myocardial viability, cardiomyopathies, congenital heart disease, and ischemic heart disease. Despite its advantages, the adoption of CMR remains uneven across regions due to barriers such as cost, lack of trained personnel, limited scanner availability, and insufficient awareness of its clinical utility [1].

These barriers—particularly the high costs of CMR scanners and their maintenance—significantly limit accessibility in low- and middle-income countries. A recent worldwide survey by Sierra-Galan et al. [2] confirmed that limited access to scanners and high costs are common obstacles for both high- and low-volume centers. While competing technologies tend to pose greater challenges for high-volume centers, inadequate referral practices emerge as a more critical barrier for low-volume centers.

Efficient CMR has been identified as a crucial factor in overcoming these barriers and increasing patient access to CMR. The SCMR has been actively raising awareness and providing tools to achieve this goal. A 30-minute CMR exam strategy was proposed in an SCMR white paper led by Raman et al. [3]. Currently, an elite group of researchers, led by Michael Markl and Vanessa Ferreira, SCMR President and Secretary-Treasurer, respectively, is developing detailed recommendations for efficient CMR, including measures to evaluate its impact in the field.

Improving patient access to CMR can be seen as the ultimate goal, enabling more precise diagnosis and better patient prognosis. Beyond CMR efficiency, other critical factors must be carefully analyzed to identify opportunities and set priorities for action, ensuring progress toward the overarching goal of patient accessibility.

My strategic priorities for SCMR in 2025 are focused on advancing accessibility and include:

1. Affordability: Develop innovative financial models to support CMR implementation, including partnerships with industry and non-profit organizations. Promote the development and adoption of low-field magnetic resonance imaging scanners, which are likely to be more cost-effective. Emphasize the use of non-contrast protocols and streamlined operational workflows to minimize staffing requirements
2. Training and education: SCMR's primary mission prioritizes global initiatives, including on-demand e-learning courses, regional workshops, mentorship programs, and collaborations with geographically distributed key institutions serving as regional training hubs worldwide. To achieve key educational milestones, we are concentrating our efforts in 2025 to ensure the success of the SCMR 2026 meeting in Rio de Janeiro—the first SCMR Annual Scientific Meeting in the Society’s history to be held outside the United States and Europe (Fig. 1).

**Fig. 1:**
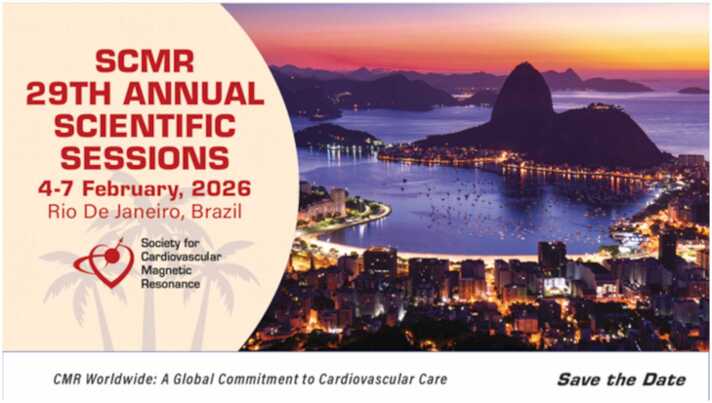
Banner and Theme for the SCMR 29th Annual Scientific Sessions, in Rio de Janeiro, Brazil.
3. Certification and credentialing: Expanding access to SCMR certification programs, subsidized fees and scholarships to low- and middle-income countries [4]. Training and certifying new CMR practitioners globally, with a focus on underrepresented regions, is a key part of our plan for the year.
4. Research: Promote research focused on the cost-effectiveness and clinical impact of CMR in diverse populations, with a strong emphasis on supporting early-career researchers and investigators from low- and middle-income countries. Enhance the global CMR Registry to enable data sharing and support large-scale studies. Foster the creation of high-quality publications and guidelines that underscore the clinical and economic value of CMR [5].
5. Awareness: Continue targeted campaigns directed at policymakers, health care providers, and patients, emphasizing a patient-centric approach to demonstrate the critical role of CMR in enhancing patient outcomes [6]. A key challenge lies in securing commitments from countries, especially low- and middle-income nations, to integrate CMR into their national cardiovascular care strategies.
6. Technology and artificial intelligence (AI): Enhance the efficiency and accessibility of CMR workflows through AI-based tools for image acquisition, analysis, and reporting, which can reduce reliance on highly specialized expertise [7], [8]. Promote open-source AI algorithms to ensure these tools are accessible to institutions globally.
7. Collaboration and advocacy: Collaborate closely with international societies, regulatory bodies, and governments (including, the World Heart Federation, and national cardiology and radiology societies around the globe) to integrate CMR into broader healthcare initiatives, such as universal healthcare packages and national diagnostic guidelines.

We should also remark some recent accomplishments of SCMR. The updated SCMR Strategic Plan, CMR Expansion Program, and Leadership Academy were recently presented and from them many new projects and action items will be deployed (https://scmr.org/our-mission-and-vision/). This very important effort was conducted by the SCMR Board of Trustees and led by Karen Ordovas.

A revamped and extremely functional SCMR website, including a CMR Center Directory with geolocation capabilities allowing visibility for CMR centers around the World (https://scmr.org/directory/), was recently launched under the leadership of Sven Plein and Steve Leung.

Our *Journal of Cardiovascular Magnetic Resonance* under the leadership of Tim Leiner and Warren Manning, current and past Editor-in-Chief, respectively, has transitioned to Elsevier publishing platform, increasing *JCMR*'s reach and impact to new heights in the scientific community.

Through the efforts of the SCMR Board of Trustees, our CEO, and the U.S. Advocacy Committee, chaired by Ibrahim Saeed, SCMR has secured a seat in the AMA House of Delegates (HOD). This milestone places SCMR alongside organizations such as the American College of Radiology, the American College of Cardiology, the American Society of Echocardiography, the Society of Nuclear Cardiology, and the Society of Computed Tomography. SCMR's membership in the AMA HOD provides a critical platform to influence key policies and payment decisions in the United States.

In his SCMR President’s page in *JCMR*, Scott Flamm wrote in 2011: “As an international Society, we have for years pursued a more global presence, though one that remained relatively U.S., Europe, and Japan-centric… increasing activity from our Latin American group has led them to request that the Society consider holding an annual meeting in Latin America (not a question of if, but how soon)” [9]. Today, we can finally answer that question: the SCMR Annual Scientific Meeting will take place from February 4–7, 2026, in the vibrant city of Rio de Janeiro, Brazil, known as the “Marvelous City.” This long-anticipated milestone began years ago with the vision and support of SCMR leaders, such as Gerald Pohost and João Lima, alongside representatives from Latin America, including Lillia Sierra-Galan, Erasmo de La Peña-Almaguer, and Juliano Lara Fernandes. Their collective efforts, jointly with those of many others, have now brought this vision to fruition, marking an exciting new chapter for our Society.

SCMR has achieved numerous additional milestones, too many to detail here, each reflecting the dedication and contributions of countless individuals. The recent hiring of Sophie Squarta as our Executive Director, alongside the exceptional leadership of our CEO, Chiara Bucciarelli-Ducci, and the Veritas team, has been instrumental in steering SCMR’s activities toward achieving our ambitious goals.

At last but not least, Claudia Prieto, Bradley Allen (Program Co-chairs), Clerio Azevedo (Abstract Chair), and Michael Markl (SCMR President) finalized an exceptional scientific program for 2025 SCMR in Washington, DC. It will be preceded by three fantastic pre-conferences (Physicians, Pediatric & Congenital, iCMR) and the SCMR-ISMRM co-provided workshop on “Cutting Edge Imaging of Cardiac Microstructure, Motion, and Strain.” We are anticipating a tremendous annual meeting that no one should miss.

I would also like to recognize our President, Michael Markl, for his remarkable work, as well as our recent past presidents—Subha Raman, Sven Plein, and Karen Ordovas—for their outstanding leadership over the past years. The rich diversity within SCMR, encompassing cardiologists, radiologists, other physicians, basic scientists, technologists, nurses, and research professionals from countless countries worldwide, is a tremendous source of pride for our society.

2025 marks a pivotal year for CMR, presenting a unique opportunity to align our collective efforts with the SCMR strategic plan. This Call to Action emphasizes prioritizing the key topics outlined here and engaging every member of the SCMR community in this shared mission. I am deeply inspired by the dedication, ingenuity, generosity, and kindness of our community, which now includes over 20,000 individuals, both active members and those connected through our messages and communications.

The future of CMR rests on our shared vision and commitment. While the challenges ahead are significant, the opportunities are equally profound. This is our moment to transform the global landscape of cardiovascular care, building on the pillars of inclusivity, innovation, and impact. Together, we can overcome barriers to global adoption and ensure that CMR transitions from being a tool for the privileged few to becoming a global standard of care that benefits patients everywhere.

## Declaration of generative AI and AI-assisted technologies in the writing process

During the preparation of this work, the author used Grammarly and ChatGPT to correct English grammar/spelling and improve readability. After using this tool/service, the author reviewed and edited the content as needed and takes full responsibility for the content of the publication.

## Declaration of competing interests

Carlos Eduardo Rochitte reports a relationship with Canon Medical Systems Corporation that includes consulting or advisory and travel reimbursement. Carlos Eduardo Rochitte reports a relationship with Pfizer Inc. that includes speaking and lecture fees. Carlos Eduardo Rochitte reports a relationship with GE Healthcare that includes speaking and lecture fees.
